# A Developmental Perspective in Learning the Mirror-Drawing Task

**DOI:** 10.3389/fnhum.2016.00083

**Published:** 2016-03-02

**Authors:** Mona Sharon Julius, Esther Adi-Japha

**Affiliations:** ^1^School of Education, Bar-Ilan UniversityRamat-Gan, Israel; ^2^Gonda (Goldschmied) Multidisciplinary Brain Research Center, Bar-Ilan UniversityRamat-Gan, Israel

**Keywords:** mirror-tracing, motor skill learning, motor-control, consolidation, long-term memory

## Abstract

Is there late maturation of skill learning? This notion has been raised to explain an adult advantage in learning a variety of tasks, such as auditory temporal-interval discrimination, locomotion adaptation, and drawing visually-distorted spatial patterns (mirror-drawing, MD). Here, we test this assertion by following the practice of the MD task in two 5 min daily sessions separated by a 10 min break, over the course of 2 days, in 5–6-year-old kindergarten children, 7–8-year-old second-graders, and young adults. In the MD task, participants were required to trace a square while looking at their hand only as a reflection in a mirror. Kindergarteners did not show learning of the visual-motor mapping, and on average, did not produce even one full side of a square correctly. Second-graders showed increased online movement control with longer strokes, and robust learning of the visual-motor mapping, resulting in a between-day increase in the number of correctly drawn sides with no loss in accuracy. Overall, kindergarteners and second-graders producing at least one correct polygon-side on Day 1 were more likely to improve their performance between days. Adults showed better performance with improvements in the number of correctly drawn sides between- and within-days, and in accuracy between days. It has been suggested that 5-year-olds cannot learn the task due to their inability to detect and encapsulate previously produced accurate movements. Our findings suggest, instead, that these children lacked initial, accurate performance that could be enhanced through training. Recently, it has been shown that in a simple grapho-motor task the three age-groups improved their speed of performance within a session and between-days, while maintaining accuracy scores. Taken together, these data suggest that children’s motor skill learning depends on the task’s characteristics and their adopting an efficient and mature performance strategy enabling initial success that can be improved through training.

## Introduction

Children are often thought to have superior skill learning abilities compared with adults. This notion has been invoked in relation to “critical” early life periods in several domains (e.g., language, Johnson and Newport, 1989; visual stereopsis, Blake and Hirsch, 1975; Packwood and Gordon, 1975). Some studies support this notion (e.g., performance of older children vs. adults on the probabilistic sequence learning task, Fischer et al., 2007; Janacsek et al., 2012; Nemeth et al., 2013). However, most laboratory studies fail to support this notion, and report an age advantage in learning of skills such as auditory temporal-interval discrimination, locomotion adaptation, applying a linguistic rule, deterministic sequence learning, and drawing visually-distorted spatial patterns (mirror-drawing (MD); e.g., Ferrel-Chapus et al., 2002; Thomas et al., 2004; Ferman and Karni, 2010; Vasudevan et al., 2011; Lejeune et al., 2013; Hodel et al., 2014). An age advantage was most frequently reported for learning within a session, but also between consecutive practice days (Huyck and Wright, 2011). One of the tasks young children failed to learn was the MD task (Ferrel-Chapus et al., 2002).

The MD task has been used in the study of skill learning since 1910 (e.g., Starch, 1910; Clinton, 1930; Ballard et al., 1993; Voderholzer et al., 2011). In this task, participants are required to trace a shape (commonly a polygon, e.g., a star, diamond, square or a triangle) and stay within the boundaries of a double line, while only seeing an inverted reflection of their hand through a mirror. Mirror learning reflects the formation of new associations between vision—rotated by 180°—and arm movement (Edelstein et al., 2004; Miall and Cole, 2007).

In motor skill-learning tasks, initial performance presumably reflects controlled processes, such as trial and error and adaptation of performance solutions, which mature with age. Later performance reflects selection of a given task solution mode and its optimization as a function of repetition (Anderson, 1982; Logan, 1988; Chein and Schneider, 2005; Adi-Japha et al., 2008; Roebers and Kauer, 2009). It has been suggested that adults adapt within a few trials to the mirror inversion because of their explicit bidirectional visuo-motor awareness of space (enabling efficient coding of visual information into movement in opposite directions) vs. unidirectional awareness in younger children, and because of their better online control of movement. The shift in visuo-motor awareness and movement control occurs at about 8 years of age, and is established at about age 11 (Ferrel et al., 2001; Ferrel-Chapus et al., 2002). On a diamond-shape MD task, in which visual feedback was rotated by 180° and appeared on a computer screen, the performance of 5-year-old children was characterized by direction changes within polygon-sides, even after many repetitions (Ferrel-Chapus et al., 2002). It has been suggested that 5-year-olds, unlike 7-year-old children, cannot learn the task because they “cannot detect accurate movements and reproduce the same programming for the next movements” (Ferrel-Chapus et al., 2002, p. 515). These findings stand in sharp contrast to 5-year-olds’ successful learning of a recently introduced simple grapho-motor task, the Invented Letter Task, in which direct visual feedback is afforded (Julius and Adi-Japha, 2015).

Five-year-olds were not the only age-group to show difficulties in learning the MD task. Ferrel-Chapus et al. (2002) compared visuo-manual coordination of children aged 5-, 7-, 9-, and 11-years and adults in mirror tracing a diamond. Only the 11-year-olds reached a performance level similar to that of adults within nine repeats, a finding recently replicated by Finn et al. (2016). Like the 5-year-olds, the 7-year-olds performed fast, ballistic movements, increasing their velocity from trial to trial, while a large number of pauses accompanied their movement. However, 7-year-olds showed less directional changes than 5-year-olds, while producing a similar number of polygon-sides. This reflected performance of some polygon-sides without directional changes. Lejeune et al. (2013) also studied age related differences in an MD task in children aged 7 and 10 years, and in adults, but used a triangle. In this task, four blocks of three trials were administered to the participants. Their findings largely replicated the findings of Ferrel-Chapus et al. (2002), indicating significant age-related differences in speed of performance, and in the number of errors produced. Lejeune et al. (2013) reported that all three age groups learned the MD task.

Studies testing the formation of visual-manual associations while adapting to other experimental conditions, also report age-advantages by which younger children adapt at a slower rate and with greater performance variability (Konczak et al., 2003; Contreras-Vidal et al., 2005; Bo et al., 2006; Kagerer and Clark, 2014). For example, when visual feedback for straight lines (in a center-out task) was rotated at 45°, 4-year-olds produced movements with the highest variability, adapting less well than 6- and 8-year-olds (Contreras-Vidal et al., 2005).

To the best of our knowledge, no study has tested between-day performance developmentally, using the MD task. Post-training performance has been tested, but only in a specific age group: children aged 10–13 years. Specifically, Prehn-Kristensen et al. (2009) tested 12 h post-training performance and Vicari et al. (2005) tested 24 h post-training performance. Both studies showed improvements in performance following the training session. For example, Vicari et al. (2005) tested the MD task in four 10-min sessions, the last session taking place the day after the initial testing. Typically developing children showed between-session improvements in both speed and accuracy during the initial training day, and a larger increase 24 h post-training. Between-session improvements were assessed while comparing performance following session completion. The current study employed a similar design.

Trial-to-trial assessment of the MD task shows performance loss between the last trial of a previous session and the first trial of the next session in adults (Snoddy, 1926) and in children (Prehn-Kristensen et al., 2009). It has been suggested that the consolidation of task-related memories amalgamates the fine-tuning motor process needed for task initiation with the memory trace, resulting in a decrease in this performance loss with practice (Buitrago et al., 2004). However, the effect of sleep dependent consolidation processes seems to differ by task performance level, being maximal at intermediate levels (Stickgold, 2009). Consolidation effects may therefore depend on task repetitions and age (Wilhelm et al., 2012). These data suggest that next-session performance is affected by many factors, and that the rate of increase in performance following a next-session, tested within a day and between days on the MD task, may differ between children in different age-groups, and between children and adults.

### The Current Study

In the current study, 5–6-year-old kindergarten children, 7–8-year-old second-graders, and young adults practiced the MD task for two sessions on two consecutive days. We aimed to investigate why it is that young children do not learn the MD task. It is not clear whether: (A) They cannot produce any correct polygon-sides, and therefore do not have an initial correct model to repeat and presumably optimize; or (B) Initial successful production is not repeated, as suggested by Ferrel-Chapus et al. (2002). We further tested several kinematic measures to characterize differences in online control of movement between age groups. Repetitions of MD production were tested over two consecutive training days because within a training day, learning could occur but not lead to performance gains. It has been shown that learning following repetition may be evident only when demonstrated in between-day improvement (Huyck and Wright, 2011; Soderstrom and Bjork, 2015).

Following the difficulties experienced by 5-year-olds in producing strokes without directional change, as described by Ferrel-Chapus et al. (2002), and following the success of 7-year-olds on the MD task described by Lejeune et al. (2013), we hypothesized that mirror learning would mature with age into adulthood.

Furthermore, Ferrel-Chapus et al. (2002) made a distinction between the 7-year-olds who were able to correctly produce one or more segments of the MD shape without directional changes, and the 5-year-olds who were not able to do so even after many repetitions. We hypothesized that improvement on the MD task in terms of correct polygon-sides requires some initial correct performance experience (as in the case of the 7-year-olds). To test this notion, we compared improvement in performance between those children who produced correct sides during initial training, and those who did not.

Specifically, we hypothesized that:

1. Kindergarten children aged 5–6- years would not be able to learn the MD task because they would hardly produce any correct polygon-sides.
2. Production kinematics would differ between the two younger age groups, and 7–8-year-olds would show better online control of movement.
3. Second-grade students aged 7–8 years would be able to perform the MD task, and with training, would improve more than 5–6 year-olds kindergarteners because they would learn the MD visuo-motor association with repeats.
4. Adults would learn the MD task better than second-grade students, in terms of speed and accuracy.
5. Only children who could produce correct polygon-sides on initial training would improve their performance following subsequent training.

## Materials and Methods

### Participants

Full data were acquired for 58 of 60 participants recruited to participate in the study. These included 19 kindergarten children (9 boys, 10 girls), aged 5 years 7 months, to 6 years 8 months (*M* = 6.17 years, *SD* = 0.33); 19 second-grade children (10 boys, 9 girls), ranging in age from 7 years 6 months, to 8 years 11 months (*M* = 8 years, *SD* = 0.43); and 20 young adults (10 men, 10 women), aged 19 years 1 month to 29 years 5 months (*M* = 23.92 years, *SD* = 3.01). One kindergartener did not want to take part in the second session of the first day. One second-grader did not want to take part in the second day of the study. Participants were recruited from centrally located regions in Israel, with medium-high socio-economic status. Israeli Ministry of Education approval of the study was received (approval number: 10.32/235/2010, 10.32/514/2011), and parents of children signed Ministry of Education consent forms. Adults signed a university-standard consent form. According to parental reports, the children recruited for the study did not have any known neurological conditions or sleep disorders. Furthermore, kindergarten teachers, as well as school teachers, identify children at risk for developmental delay in the first 3 months of the school year (Ministry of Education, 2007). These children were not included. All participants were right-handed, based on the Hand Dominance Questionnaire (Oldfield, 1971). The parents of the younger participants answered 10 age-matched questions on the questionnaire for their children (RH: range 7–10, *M* = 8.75, *SD* = 1.16; range 7–10, *M* = 8.75, *SD* = 0.97, kindergarten and second-grade, respectively). Adults answered all 14 questions (range 10–13, *M* = 12.05, *SD* = 0.89).

The participants of the current study were part of a larger study focusing on the association between motor skill learning (assessed using the Invented Letter Task) and academic achievements (Julius, 2015). The participants (except for one adult) were assessed on additional measures, including tests of visuo-motor skills known as predictors of handwriting in children (Feder and Majnemer, 2007). These tests are reported below.

### Procedure

All sessions were conducted in a quiet room, over two consecutive days. Token rewards such as school supplies, were distributed to the children at the end of each day. In the instance of the children, handedness and visuo-motor skills were tested in sessions separate from the MD task. In the adults, these were assessed following the last MD session.

### The Mirror Drawing Task

For the MD task we employed the shape of a square that does not require diagonal lines, because diagonal lines are considered more complex to produce (Gullaud-Toussaint and Vinter, 2003; Feder and Majnemer, 2007). The MD task apparatus consisted of a box that was constructed to hold a mirror allowing the participants to see the target page, but not to see their hand. Based on our preliminary trials, and bearing in mind the age-dependent abilities of the younger participants in this study, we adopted a square shape as our MD task. Following Vicari et al. (2005), we used timed sessions, but shortened the length of the session compared to Vicari et al. (2005) from 10-min sessions to 5 min per session. The task was performed over two consecutive days; two 5-min sessions, 10 min apart, were held on two consecutive days. Participants traced the outline of a square between two lines while seeing their hand only through a mirror. They were not able to see the square directly, as the MD apparatus blocked their vision. The length of each side of the square was 11.5 cm, and the distance between the inner and outer contour was 0.9 cm. Participants were instructed to complete as many squares as possible during the allotted time while staying within the double line—the inner and outer parameters of the square. Upon completion of a square, another sheet of paper was placed in the MD apparatus by the experimenter. On several occasions, the younger children requested a new sheet before completing a square.

#### Coding

Following the observation by Ferrel-Chapus et al. (2002) regarding the differences between 5- and 7-year-olds’ correct movement-segments, only correct movement-segments of at least one polygon-side length (i.e., one side of a square) were analyzed. Thus, similar to Vicari et al. (2005), we measured speed according to the number of correctly produced polygon-sides in each session (rather than the overall number of polygon-sides). Correct polygon-sides were defined as sides of the square produced with no lines that crossed over either the inner or the outer parameter of the square, and no pen-lifts, with at least one correctly performed corner turn. The ratio of incorrect polygon-sides to the total number of polygon-sides served as the main error measures of analyses. Interrater reliability (ICC measure), calculated for 12 participants (4 in each age group, overall 20% of the data), was 0.95 (*p* < 0.001).

In order to characterize the MD solution strategy used by the two children’s groups, additional kinematic measures were coded. These included the total number of sides of a square completed (correct and incorrect polygon-sides), and all pathway line crossings (either escaping from between the double line, or entering the double line) committed per shape. Following Vicari et al. (2005), an error ratio measure (overall number line crossings divided by the number of polygon-sides produced, per session) was calculated. Reversals (direction change) and pen-lifts per shape were also coded (Ferrel-Chapus et al., 2002; Gullaud-Toussaint and Vinter, 2003), and their ratio to the total number of polygon-sides produced was calculated.

### Visuo-Motor Skills

The Beery Buktenica developmental test of visuo-motor integration (Beery-VMI) is frequently administered to evaluate the quality of abilities that may underlie problematic handwriting. The main idea is that the acquisition and preservation of readable handwriting requires one to be able to recognize shapes; to use vision to control arm, hand, and finger movements; and to coordinate the movements of these effectors accurately. Three subtests of the Beery-VMI were developed to test these abilities in children between 2- and 17-years. The test is the norm referenced for American children from 2- to 18-years.

The visual perception subtest (VP) measures whether children can discriminate geometric figures. The visuo-motor integration subtest (VMI) is used to assess children’s ability to copy similar geometric figures, while the motor coordination subtest (MC) requires children to draw figures in between lines (Beery et al., 1997). All three tests use 27 geometric figures, starting with simple figures and ending with more complex ones. All children participating in the study had a standardized VMI score ≥85, apart from one second-grader who had a standard score of 76. Adults, evaluated using 18-year-olds standards, scored 86 or above. Performance below the 5th percentile (standardized score <75) is appropriate for the definition of a motor impairment which impacts children’s daily life (Lingam et al., 2009).

The MC-subtest was used in the current study as a covariate of motor performance. The MC-subtest was preferred because it has the same visual features as the MD task, but it enables normal visual feedback. In the MC-subtest, the participant draws a line within each of the 24 figures. The line is drawn in a gap between an inner and an outer borderline (as in the MD task). Two dots define the beginning and the end of the line (“draw a line from the black dot to the gray dot. Try to stay inside the track”). Completion time is within a maximum of 5 min. Participants were not allowed to use an eraser. All correctly drawn figures (i.e., between the lines) were scored. Standardized MC scores were: *M* = 92.70, *SD* = 17.23; *M* = 84.11, *SD* = 9.54; *M* = 92.42, *SD* = 7.54, for kindergarteners, second-graders and adults, respectively, range = 76–115; raw scores were: *M* = 13.15, *SD* = 3.51; *M* = 14.70, *SD* = 2.41; *M* = 25.37, *SD* = 1.86, respectively. Analysis of Variance (ANOVA) indicated a significant group differences in raw scores *F*_(2,55)_ = 117.65, *p* < 0.001, that emerged because adults had much higher scores than the two younger groups (Bonferroni, *p*’s < 0.001) that did not differ significantly (*p* = 0.22). Differences in standardized between these groups were insignificant as well *F*_(1,36)_ = 1.82, *p* = 0.08.

### Analytic Plan

The aim of the current study was to understand why 5-year-olds (and possibly some 7-year-olds) do not learn the MD task. To this end, we compared the performance of three age-groups on the number of correctly performed polygon-sides, and the ratio of incorrect polygon sides. Differences between the three age-groups on the measures of the number of correct polygon-sides produced and the ratio of incorrect polygon-sides were studied using a 2 (Day) × 2 (Session) × 3 (Group) Analysis of Variance for repeated measures (rmANOVA). The main effects were reported, but where these were followed by interactions, only the interactions were explained. Based on the hypotheses that 5–6-year-old kindergarten children would not show learning, and that 7–8-year-olds second-graders would learn, but less so than adults, significant Group main effects were followed by comparing the 5–6- to the 7–8-year-olds (first contrast), and by comparing the 7–8-year-olds to adults (second contrast). Similarly, interactions were followed by using interaction contrast analysis. In case of violations of equality of variances appropriate testing procedures were used, correcting for degrees of freedom.

Because learning may be affected by motor ability, the rmANOVAs were repeated with the raw scores of the Beery MC-subtest as a covariate. To differentiate between the effects of age-group and motor ability on learning, one condition is that the age-group variable and MC-subtest scores must be independent before entering the analyses. As indicted in “Visuo-Motor Skills” Section above, this held true only for the raw (and standardized) scores of the two younger age-groups. The second condition is that the age-group × MC-subtest interaction with respect to the dependent variable be insignificant (Miller and Chapman, 2001). We therefore restricted the analysis with the MC-subtest used as a covariate to the two younger age-groups, and reported the analyses only after verifying a non-significant interaction term in a preliminary analysis (i.e., a non-significant group by MC-subtest interactions when the MC-subtest is incorporated into the analysis). Analyses done with raw scores used as a covariate were then repeated with standardized scores used as a covariate.

In order to characterize developmental changes in performance strategy (from ballistic to online movement control), we analyzed the total number of polygon-sides, and the ratios per polygon-side of pen-lifts, pathway line-crossing errors, and reversals. These analyses pertained only to the two younger age groups, and were carried out between- as well as within-groups.

Parametric as well as non-parametric tests were used to test the hypothesis that of the 38 children, only children who could produce correct polygon-sides on initial training would improve their performance following subsequent training. The definition of what constituted initial training and subsequent training was based upon the emergence of learning gains in second-graders vs. kindergarteners. We tested improvement differences on subsequent training between those children who did produce correct polygon-sides on the initial portion of the training and those who did not. The non-parametric analysis was performed using Sign tests and a *z*-ratio test. The Sign test compared subsequent-training improvement among those who did/did not produce correct sides in initial training. The *z*-ratio test compared the proportion of subsequent-training improvement between these two groups. Our hypothesis would be supported if the *z*-ratio test were to find that among those children producing correct sides during initial training, there would be a significant larger proportion of children who improved vs. those who did not produce correct polygon-sides. The parametric analyses compared the magnitude of improvements between these two groups.

## Results

**Figure 1:**
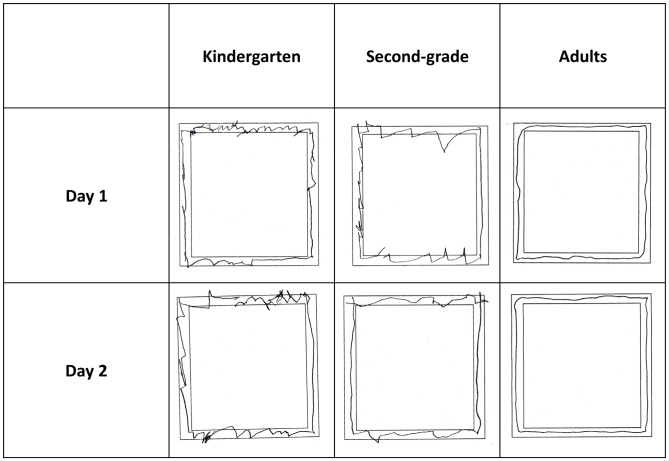
**Examples of mirror drawing (MD) production by age group: 5–6-year-old kindergarteners, 7–8-year-old second-graders, and young adults**.

**Figure 2:**
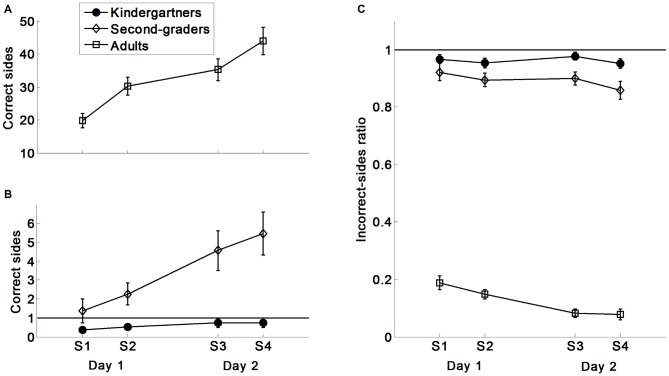
**Main outcome measures: the number of correct sides and the ratio of incorrect sides/total sides for children and adults. (A)** Correct sides in adults. **(B)** Correct sides in children. **(C)** Incorrect-sides ratio.

Examples of MD production by age group are provided in Figure 1. Figure 2 presents the number of correct polygon-sides, and the ratio of incorrect polygon-sides to the total number of polygon-sides for each age group: 5–6-year-old kindergarten children, 7–8-year-old second-graders, and young adults.

### Number of Correct Polygon-Sides

The 2 (Day) × 2 (Session) × 3 (Group) rmANOVA pertaining to the number of correct polygon-sides indicated a main effect of Group *F*_(2,55)_ = 117.27, *p* < 0.001, *η^2^* = 0.81, and a main effect of Day *F*_(1,55)_ = 32.71, *p* < 0.001, $\eta_{p}^{2}$ = 0.37, modulated by a Group × Day interaction *F*_(2,55)_ = 17.03, *p* < 0.001, $\eta_{p}^{2}$ = 0.38. The group contrast comparisons revealed that the between-day improvement (the difference in improvement during Day 2 vs. during Day 1) was greater for the second-graders than for the kindergarteners *F*_(1,19.74)_ = 12.09, *p* < 0.01, and greater for the adults than for the second-graders *F*_(1,22.10)_ = 13.35, *p* < 0.01. Only the second-grade students and adults improved between days (*F*_(1,18)_ = 15.31, *p* < 0.01; *F*_(1,19)_ = 24.29, *p* < 0.001, respectively).

The rmANOVA further indicated a main effect of Session *F*_(1,55)_ = 61.62, *p* < 0.001, $\eta_{p}^{2}$ = 0.53, that was modulated by a Group × Session interaction *F*_(2,55)_ = 46.23, *p* < 0.001, $\eta_{p}^{2}$ = 0.63. The group contrast comparisons indicated that the improvement from Session 1 to Session 2 was greater in the adults than in the second-graders (*F*_(1,26.71)_ = 42.46, *p* < 0.001) because only the adults improved between sessions, *F*_(1,19)_ = 65.19, *p* < 0.001. No other interactions emerged.

These data suggest a different rate of improvement between groups. Kindergarten children did not improve their performance as a result of training. Second-graders gained between days more than kindergarteners. Adults gained more than second-graders between sessions and between days.

### The Ratio of Incorrect Polygon-Sides

The rmANOVA pertaining to the ratio of incorrect polygon-sides (number of incorrect polygon-sides/total number of polygon-sides) indicated a main effect of Group *F*_(2,55)_ = 941.76, *p* < 0.001, *η^2^* = 0.97, and a main effect of Day *F*_(1,55)_ = 12.07, *p* < 0.001, $\eta_{p}^{2}$ = 0.18, modulated by a Group × Day interaction *F*_(2,55)_ = 6.41, *p* < 0.01, $\eta_{p}^{2}$ = 0.19. The group contrast comparisons indicated that adults improved between days significantly more than second-graders (*F*_(1,35.58)_ = 4.54, *p* < 0.05), because only the adults improved between days, *F*_(1,19)_ = 23.43, *p* < 0.001.

The rmANOVA further indicated a main effect of Session *F*_(1,55)_ = 8.61, *p* < 0.01, $\eta_{p}^{2}$ = 0.14, whereby the performance during the second session was more accurate than during the first. No other interactions emerged.

### Developmental Differences Between Kindergarteners and Second-Graders Beyond Motor-Coordination Ability

It may be suggested that group differences in learning partially reflect differences in motor ability, rather than in learning, *per se*. To test this possibility, the above analyses were repeated with the raw (and standardized) scores of the MC-subtest of the Beery-VMI as a covariate in analyses that pertained to the two groups of children. In the MC-subtest, participants draw figures between a double-line (see “Materials and Methods” Section), while afforded with visual feedback. The results of the MC-subset did not differ significantly between kindergarteners and second-graders. A preliminary analysis indicated that group × MC-subtest was insignificant in the analyses of the number of correct polygon-sides and the ratio of incorrect polygon-sides.

The rmANOVAs pertaining to the number of correct polygon-sides, in the presence of the raw MC-subtest as a covariate, indicated a significant effect of Group (*F*_(1,35)_ = 13.87, *p* < 0.01, $\eta_{p}^{2}$ = 0.28). The Group main effect was modulated by a Group × Day interaction (*F*_(2,53)_ = 12.14, *p* < 0.01, $\eta_{p}^{2}$ = 0.26), because only second-graders improved between days. The analyses pertaining to the ratio of incorrect polygon-sides indicated only a main effect of group (*F*_(1,35)_ = 7.81, *p* < 0.01, $\eta_{p}^{2}$ = 0.18) due to the better performance of the second-graders. The same pattern of results was received when the standardized MC-subtests were used as a covariate. These analyses indicate that differences between kindergarteners and second-graders could not be fully accounted for by differences in MC.

### In Depth Analysis of the Differences Between Kindergarteners and Second-Graders

**Figure 3:**
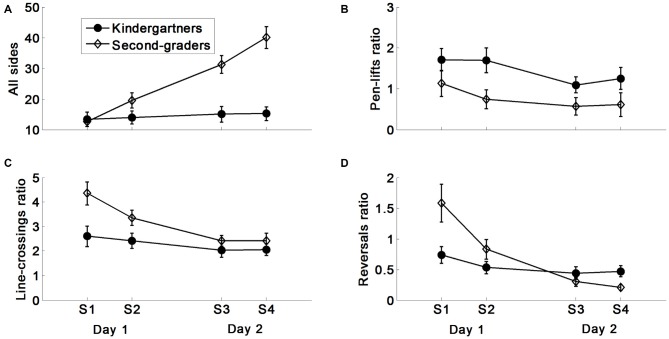
**Children’s production characteristics. (A)** Overall number of sides. **(B)** Pen-lifts ratio. **(C)** Line-crossings ratio. **(D)** Reversals ratio. The ratios were computed as number per side.

In order to test the source of the difference in the learning profile between kindergarteners and second-graders, a detailed kinematic analysis was carried out between age-groups and within age-groups. Four additional measures are presented: the total number of polygon-sides produced, the ratios of pen-lifts, pathway line-crossing errors, and reversals per polygon-side (Figure 3). It should be noted that for all analyses the Group × Day interactions, whenever appearing, were retained even when the Beery MC-subtest (either raw or standardized) was used as a covariate. For simplicity, we report here the analysis without the covariate.

#### Total Polygon-Sides

The between group analysis of the total number of polygon-sides indicated a main effect of Group, *F*_(1,36)_ = 13.76, *p* < 0.001, *η^2^* = 0.28. Furthermore, the analysis indicated a main effect of Day *F*_(1,36)_ = 56.88, *p* < 0.001, $\eta_{p}^{2}$ = 0.61, modulated by a Group × Day interaction *F*_(1,36)_ = 42.07, *p* < 0.001, $\eta_{p}^{2}$ = 0.54. There was also a main effect of Session, *F*_(1,36)_ = 19.71, *p* < 0.01, $\eta_{p}^{2}$ = 0.36, modulated by a Group × Session interaction, *F*_(1,36)_ = 16.77, *p* < 0.001, $\eta_{p}^{2}$ = 0.31. The interactions emerged because only second-grade students increased the total number of polygon-sides produced between days and between sessions (*F*_(1,18)_ = 80.30, 33.46, respectively, *p*s < 0.001). No other interactions emerged. Significant interactions were retained even when the MC-subtest was added as a covariate.

#### Pen Lifts

The analysis of the rate of pen-lifts per polygon-sides indicated an overall higher rate of pen-lifts in kindergarteners, *F*_(1,36)_ = 4.33, *p* < 0.05, *η^2^* = 0.11. The analysis further indicated that the number of pen-lifts was lower on the second day (i.e., when performance was summed over the two sessions) *F*_(1,36)_ = 10.59, *p* < 0.01, $\eta_{p}^{2}$ = 0.23, for both groups. No other main effects or interactions emerged. The Day main effect was not significant after the MC-subtest was added as a covariate.

#### Line-Crossing Errors

The analysis of the rate of line-crossing errors indicated a main effect of Group, *F*_(1,36)_ = 5.17, *p* < 0.03, *η^2^* = 0.13. Furthermore, the analysis indicated a main effect of Day *F*_(1,36)_ = 22.47, *p* < 0.001, $\eta_{p}^{2}$ = 0.38, modulated by a Group × Day interaction *F*_(1,36)_ = 6.00, *p* < 0.02, $\eta_{p}^{2}$ = 0.14. The interaction emerged because only second-grade students decreased their line-crossing error-rate between days (*F*_(1,18)_ = 25.28, *p* < 0.001). The rmANOVA further indicated a main effect of Session, *F*_(1,36)_ = 4.57, *p* < 0.05, $\eta_{p}^{2}$ = 0.11, modulated by a Day × Session interaction, because line-crossing error-rate significantly decreased only on Day 1, *t*_(38)_ = 2.93, *p* < 0.01, and to a much lower extent on Day 2. No other interactions emerged. Only the Group × Day interaction remained after the MC-subtest was added as a covariate.

#### Reversals

Unlike out-of-line errors and pen-lifts, reversals were not defined as errors. They primarily indicate to what extent children learned the visual-motor map. The analysis of reversals indicated no overall group differences. In contrast, all other main effects and interactions emerged as significant, including the triple interaction of Day × Session × Group, *F*_(1,36)_ = 4.86, *p* < 0.04, $\eta_{p}^{2}$ = 0.12 (for all other mean effects and interactions *F*_(1,36)_ = 6.10, *p*s < 0.02). The triple interaction emerged because on Day 1 Session 1, second-graders began with a higher rate of reversals than kindergarteners, *t*_(36)_ = 2.67, *p* < 0.02. However, the rate of second-graders’ reversals decreased across sessions (*F*_(1,18)_ = 18.17, *p* < 0.001), while that of kindergarteners was maintained. Finally, in the last session (Day 2 Session 2), second-graders had a lower rate of reversals than kindergarteners, *t*_(36)_ = 2.57, *p* < 0.02. These data suggest that only second-grade students learned the visual-motor map. It should be noted that when the Beery MC-subtest was used as covariate the triple interaction decreased, and was at the *p* = 0.066 level only.

### Within-Group Analyses

Tables 1, 2 include a detailed analysis of the learning within each of the age-groups (these tables match Figures 2, 3). Table 1 indicates that across days, kindergarten children did not improve their performance significantly, in terms of either correct polygon-sides, or the rate of incorrect polygon-sides, while second-grade students significantly improved in the former, with no decrease in the latter.

**Table 1:** **Within-group comparison of correct sides and incorrect sides ratio, across sessions and days**.

	Between days: (*D*_1_*S*_1_ + *D*_1_*S*_2_) − (*D*_2_*S*_1_ + *D*_2_*S*_2_)	Between sessions: (*D*_1_*S*_1_ + *D*_2_*S*_1_) − (*D*_1_*S*_2_ + *D*_2_*S*_2_)	Day × Session	Within day 1: *D*_1_*S*_2_ − *D*_1_*S*_1_	*D*_2_*S*_1_ − *D*_1_*S*_2_	Within day 2: *D*_2_*S*_2_ − *D*_2_*S*_1_
	*F* day	*F* session	*F* interaction	*t*	*t*	*t*
**Correct sides**
Kindergarten	2.57	0.32	0.81	1.37	1.00	0.00
Second-grade	15.31***	2.88	0.01	1.44	2.55*	1.04
Young adults	24.29***	65.19***	0.72	7.84***	2.20*	4.73***
**Error rate: incorrect sides/total sides**
Kindergarten	0.68	2.65	1.15	0.91	1.24	2.42*
Second-grade	1.65	3.46	0.12	0.88	0.28	1.65
Young adults	23.43***	2.47	1.18	1.90	3.44**	0.08

*p < 0.05, **p < 0.01, ***p < 0.001. Kindergarten, n = 19; Second-grade, n = 19; Young adults, n = 20. D = Day. S = Session.

**Table 2:** **Within-group comparison of kinematics measures across sessions and days**.

	Between days: (*D*_1_*S*_1_ + *D*_1_*S*_2_) − (*D*_2_*S*_1_ + *D*_2_*S*_2_)	Between sessions: (*D*_1_*S*_1_ + *D*_2_*S*_1_) − (*D*_1_*S*_2_ + *D*_2_*S*_2_)	Day × Session	Within day 1: *D*_1_*S*_2_ − *D*_1_*S*_1_	*D*_2_*S*_1_ − *D*_1_*S*_2_	Within day 2: *D*_2_*S*_2_ − *D*_2_*S*_1_
	*F* day	*F* session	*F* interaction	*t*	*t*	*t*
**Overall number of sides**
Kindergarten	1.04	1.43	0.10	0.45	0.58	0.11
Second-grade	80.30***	33.46***	0.77	4.95***	5.82***	4.34***
**Pen-lifts ratio**
Kindergarten	5.87*	0.35	0.58	0.18	2.89**	0.91
Second-grade	4.87*	1.53	3.82	2.25*	2.29*	0.24
**Line-crossings ratio**
Kindergarten	2.68	0.32	0.39	0.85	1.47	0.13
Second-grade	25.28***	4.26*	7.59*	3.11**	3.67**	0.65
**Reversals ratio**
Kindergarten	2.09	0.89	3.96	1.83	0.69	0.26
Second-grade	21.78***	18.17***	18.03***	4.68**	4.25**	2.25*

*p < 0.05, **p < 0.01, ***p < 0.001. Kindergarten, n = 19; Second-grade, n = 19. D = Day. S = Session.

Table 2 shows that the second-graders improved on the additional four kinematic measures within the first day, and between days. This explains, at least partially, their between-day improvement in the number of correct polygon-sides produced (Figure 2). Kindergarten children showed improvement only in the ratio of pen-lifts to polygon-sides produced between days (reduction of pen-lifts ratio while maintaining their overall number of polygon-sides produced), suggesting that similar to second-graders, these children produced longer strokes on Day 2 than on Day 1.

### Learning of the MD Task in Children Who did/did not Produce Correct Polygon-Sides on Day 1

On average, kindergarten children did not produce even one correct polygon-side, and had above 95% inaccurate polygon-sides throughout the experiment (Figure 2). Second graders had on average more than three polygon-sides correctly produced by the end of Day 1. The achievements of second-graders on Day 2 suggest that having at least one polygon-side correctly performed on Day 1 was a sufficient experience for a between-day improvement. Among the 14 second-graders who produced one or more polygon-sides on Day 1, 11 improved (by at least by one polygon-side), two maintained their performance level, and one child preformed less well on Day 2 than on Day 1 (Sign-test, *p* < 0.001).

Of the 38 children, 23 produced correct polygon-sides on Day 1. Of these 23 children, 15 improved between days, five maintained their performance, and three children performed less well on Day 2 than on Day 1 (Sign-test, *p* < 0.01). Of the 15 children who did not produce any correct polygon-sides on Day 1, only five improved between days. These data suggest that children who had at least one polygon-side correctly performed on Day 1, were more likely to improve between days than their peers (15/23, vs. 5/15, *z* = 1.9241, *p* = 0.0548). Furthermore, among the 20 children who improved their performance between days (6 kindergarteners, 14 s graders), 15 (4 kindergarteners, 11 second-graders) had at least one polygon-side correctly produced on Day 1 (Binomial, *p* < 0.05), suggesting that between-day improvers were more likely to already have some experience in correctly producing polygon-sides.

The 23 children who had correct sides on Day 1 significantly improved their performance between days, *t*_(22)_ = 3.47, *p* < 0.01, and their improvement was larger than that of their peers, *F*_(1,36)_ = 4.33, *p* < 0.05. The 15 children who did not produce any correct sides on Day 1 did not show a significant between day improvement, *t*_(14)_ = 1.83, *p* > 0.09. Both groups did not change their accuracy scores between days. These data corroborate the notion that only children who had some experience in solving the MD task on Day 1, were able to succeed more on Day 2 because of practice. The children who did not have this experience (and kindergarten on average) did not improve between days.

In order to test the difference in the learning profile *within* Day 1 between those who produced and those who did not produce correct polygon-sides, the performance of these two groups was compared on the additional four kinematic measures. The analysis indicated that both groups similarly increased their overall number of sides produced *F*_(1,36)_ = 7.61, *p* < 0.01, reduced their ratio of line-crossing, *F*_(1,36)_ = 10.53, *p* < 0.01, and of reversals, *F*_(1,36)_ = 21.44, *p* < 0.001 with no group interactions. However the children who produced correct polygon-sides on Day 1 had an overall higher number of sides produced, *F*_(1,36)_ = 7.61, *p* < 0.001, and lower ratio of pen-lifts *F*_(1,36)_ = 41.75, *p* < 0.001. These data suggest that both groups improved on some of the kinematic measures; however, the children who produced correct polygon-sides on Day1 had an overall better performance than their peers did. These children (like second-graders vs. kindergarteners on average) were able to produce longer strokes.

## Discussion

The current study tested developmental differences in within-day and between-day learning on the MD task. Three age groups were tested: 5–6-year-old kindergarten children, 7–8-year-old second-graders, and young adults. Kindergarten children produced on average less than one correct polygon-side per session, had less than 5% accurate polygon-sides produced overall, and did not improve their performance significantly throughout the experiment. The use of many oriented short segments characterized their performance. Second-grade students showed better online control of movement, enabling them to produce fewer segments per polygon-side. They showed robust learning of the visuo-motor association, accompanied by a reduction in the ratio of out-of-line errors and pen-lifts. This enabled them to produce more correct polygon-sides on Day 2 than on Day 1, while maintaining their accuracy scores (no speed-accuracy trade-off). Differences in MC between kindergarteners and second graders did not account for this performance difference between the groups. Adults produced more sides that were correct and were more accurate than the two groups of children. In the adult group, both the number of correct sides produced and accuracy scores improved between days, while the number of correctly produced polygon-sides increased within-days as well. The main difference between the age groups involved between-day improvement, which increased with age.

Overall, children producing at least one correct polygon-side on Day 1 (23/38 children) were more likely to improve their performance between days. For these children only, performance on Day 2 was better than on Day 1 in terms of correct polygon-sides produced, with no reduction in accuracy. These data corroborate the notion that initial effective training experience that involves correct task solutions is needed in order to show gains in correct performance. Kindergarten children, or those who did not produce correct-sides on Day 1, improved in some aspects of their kinematic production, thereby indicating improvement with repeats. However, this improvement did not bring about a between-day increase in the number of correct polygon-sides produced. Possibly, these children had less practice opportunities due to the use of multiple strokes per side. Fewer strokes per side may offer more opportunities for movement corrections (e.g., via direction changes). Lack of success may indicate that the task was too difficult for some of the children. Future studies may test whether explicit instructions while performing the task (e.g., try producing longer lines, try staying within the double-line) may help children to solve the task.

In line with previous developmental studies of MD learning (Ferrel-Chapus et al., 2002; Lejeune et al., 2013; Finn et al., 2016), the findings of the current study suggest that MD learning matures with age. This finding is in line with results of other motor adaptation studies (Konczak et al., 2003; Contreras-Vidal et al., 2005; Bo et al., 2006; Kagerer and Clark, 2014). However, these findings do not indicate that for all tasks motor skill learning matures with age (Dorfberger et al., 2007; Ashtamker and Karni, 2013; Adi-Japha et al., 2014). A recent study of a grapho-motor task requiring the reproduction of a simple “Invented Letter”, a dot-to-dot connecting task forming a two-segment pattern, indicated a similar learning profile within and between days in kindergarten children, second-graders, and adults (Julius and Adi-Japha, 2015). In the Invented Letter task, direct visual feedback exists. These data suggest that children’s motor skill learning depends on the task’s characteristics such task complexity and the affordance of visual feedback (Ferrel-Chapus et al., 2002). In simple tasks that do not require much attentional resources and on-line control, children improve in the same way as adults (Dorfberger et al., 2007; Adi-Japha et al., 2014; Julius and Adi-Japha, 2015), suggesting that efficient skill learning exists early on. The learning of complex tasks may require more controlled trail-and-error processes and more adaptation of performance solutions, which mature with age, in order to find an initial task solution/performance mode (Adi-Japha et al., 2008).

Furthermore, the findings of the current study show that performance indications for MD-learning in children, in terms of correctly performed sides, first emerge between days: in second-graders, performance on the second day of the study was significantly better than on the first (Vicari et al., 2005). In terms of accuracy, only adults showed significant improvement, also between days. In line with the similarity between motor and perceptual learning (Censor et al., 2012), training on different auditory perceptual tasks (Huyck and Wright, 2011, 2013) suggests late maturation of the learned skills, with indication of performance gains emerging between, rather than within, days. It has been suggested that inattention due to repeated experiences and fatigue may contribute to the finding of lack of improvement within sessions. Studies on fatigue suggest that learning could occur even after fatigue prevents any further gains in performance during acquisition. Fatigue build-up can also cause worsening in performance (Rickard et al., 2008; for a review on the difference between learning and performance, see Soderstrom and Bjork, 2015). Future studies may test whether older children and adolescents show within session MD learning.

The current study sought to test the source of difficulty kindergarten children have in MD learning, relative to older children. Kinematic analysis applied to the two groups of children studied here revealed that the kindergarteners lifted their pen more times per side, indicating that these children produced many segments. Overall, kindergarteners maintained a stable ratio of line crossings and directional changes (reversals) to polygon-sides produced. Second-graders produced fewer segments per side, but initially had more line crossings per side than kindergarteners had. These crossings emerged because of the many reversals second-graders produced due to the visual distortion. Initial rate of reversals was higher in second-graders than in kindergarteners. Furthermore, second-graders corrected their movement online by reversals, while most kindergarteners could not and preferred initiating new segments. Reversals were initially of a much larger magnitude in second-graders, but dropped with practice to a lower level than that of the kindergarteners. A drop in the rate of line crossings mirrored the drop in reversals. In both kindergarteners and second-graders, the number of pen-lifts per side was reduced across days. This indicates a decrease in the number of segments used per side, and suggests an increase in their length (covering the same trajectory length but with less segments). Possibly, the preference of kindergarteners to initiate many new segments while trying to stay within the double-line (see Figure 1) lowered their performance rate and prevented a significant increase in the overall number of sides produced (which increased insignificantly from 13 sides/session on Day 1 to 15 sides/session on Day 2).

On the whole, the kinematic analysis indicated that the 5–6-year-old kindergarten children exhibited a ballistic mode of control (rapid movements, followed by stopping for error evaluation that resulted in pen lifting). With practice, their motor control improved; therefore, they were able to reduce the number of segments used. They did not reduce their error rate, suggesting that in spite of an attempt to stay within the double-line, they repeatedly crossed the line and returned between the double-line. The 7–8-year-old second-graders in the current study also used ballistic movements, but to a lesser degree. Importantly, due to their better online movement control, second-graders were able to learn the visual-motor mapping, as indicated by the decrease in reversal rates with practice. Our results concur with the classic motor control literature, suggesting that the performance at 7 years of age is characterized by the dominance of the visual guiding system (e.g., Hay, 1978, 1979; Chicoine et al., 1992; Adi-Japha and Freeman, 2001; Contreras-Vidal et al., 2005). These results contrast with the report of MD learning made by Ferrel-Chapus et al. (2002) who concluded that 5- and 7-year-olds used a similar, ballistic strategy. The difference between the studies may be related to the between-day design of the current study enabling a longer period of learning, or to the somewhat older age of the second-graders in the current study.

We also studied production kinematics differences within the 1 day, between those who did and those who did not produce correct sides on that day (9/19 kindergarteners and 14/19 second-graders produced correct sides on the first day). The analysis indicated similar within-day learning characterized by an increase in the overall number of sides produced, and a decrease in the number of line crossings and direction changes per side. Importantly, the group of children who produced correct sides on the first day, produced more sides overall (i.e., correct and incorrect) that day, and had less segments per side (suggesting that their segments were longer). Overall, these children had more experience in correcting their production through directional changes, which possibly enabled them to learn the MD visuo-motor representation. Taken together with the kindergarteners reduction in the number of segments produced between days, these data suggest that those not producing correct sides on the first day of the study understood the MD task and tried to the solve it, but did not succeed, probably because of a lower level of online movement control.

### Limitations and Conclusions

The findings reported here must be considered within the limitations of the study. Only a small sample of participants of a very specific age-range per age group was studied. Only one simple MD shape was used that does involving diagonal lines. The kindergarten children showed a low success rate, resulting in low variability. Second graders differed in their motor profile, which may have contributed to greater performance variability in this group. Adults had an overall better performance with accompanying greater variability. Differences in performance variability may have inflated the Type I error, thereby increasing the probability for rejecting the null hypothesis. The low success-rate of the kindergarteners may also suggest that some of them did not understand the task. However, it should be noted that many children tried to peak at their hands in order to have a direct view, suggesting that these children were aware of the difficulty induced by the MD inversion. Although the children in the current study were typically developing, as reported by their parents and teachers, learning disabilities or attentional disorders may be diagnosed later. Lastly, the findings are of a correlational nature; therefore, causality may not be inferred.

Consistent with previous studies, the findings of the current study suggest that MD learning matures with age. Furthermore, similar to perceptual tasks, performance indications for learning in children first emerge between days. The findings support the notion of a minimal *correct* experience necessary for between-day improvement. In line with the literature, the findings support the notion of a qualitative difference in the underlying motor control strategy used in the MD task by kindergarten children, second-graders, and adults.

## Author Contributions

MSJ and EA-J conceived and designed the experiments, analyzed the data, and wrote the article. MSJ performed the experiments.

## Conflict of Interest Statement

The authors declare that the research was conducted in the absence of any commercial or financial relationships that could be construed as a potential conflict of interest.
